# Understanding wellbeing together with children from migrant backgrounds living in socio-economically disadvantaged neighborhoods- A participatory photovoice study

**DOI:** 10.1186/s12889-026-29910-9

**Published:** 2026-10-12

**Authors:** Rathi Ramji, Therese Sterner

**Affiliations:** 1 Department of Care Science, Faculty of Health and Society, Malmö University, Jan Waldenstromsgatan 25, Malmö, 202506 Sweden; 2 Citizen Health Research Centre, Malmö University, Malmö, Sweden; 3 Department of research, development, education and innovation, Skåne University Hospital, Lund, Sweden

**Keywords:** Child wellbeing, Socioeconomically disadvantaged neighborhoods, Migration, Community based participatory research, Photovoice, Social inclusion

## Abstract

**Introduction:**

Although Sweden provides universal child services, unequal access to supportive environments persists particularly among children with migration backgrounds. Children with migration background living in socioeconomically disadvantaged neighborhoods encounter structural and social conditions that influence their health, development, and lived experiences. Further, there is limited knowledge about how children define wellbeing in these contexts. Such knowledge is essential for understanding inequities in the social conditions that shape children’s health and wellbeing, and for informing policies aimed at reducing these inequities. Thus, this study aims to explore through a participatory process how children with migration background in socioeconomically disadvantaged neighborhoods define wellbeing.

**Methods:**

A study with qualitative participatory action research approach was carried out together with 31 children (15 boys and 16 girls) aged 10–12 with a migration background who participated in activities at three leisure time centers in one of the large cities in Southern Sweden. Data was collected for over a year through multistage focus groups guided using the photovoice method. Children documented aspects of their daily lives, created collages, and worked collaboratively to analyze and interpret insight cards. All discussions were audio recorded and synthesized with the thematic structure developed by the children.

**Results:**

Three themes described the children’s understanding of wellbeing. The first theme, feeling safe and supported, included emotional security, respectful communication from adults, and access to food, clean surroundings, and play materials. The second theme, belonging and being included, reflected the importance of fairness, acceptance, shared enjoyment, and influence in decisions affecting them. The third theme, growing and developing as a person, included opportunities to learn new skills, engage in creative activities, and develop socially and emotionally through play and group interaction.

**Conclusion:**

Children’s definitions of wellbeing were connected to the realities of growing up with a migration background in disadvantaged neighborhoods. They described wellbeing in terms of conditions not consistently available to them, although many established frameworks assume such conditions are already met. These findings highlight inequities in the social determinants that shape wellbeing of children with migration backgrounds and underscore the need for policies and interventions that address structural and environmental barriers.

## Background

Sweden was one of the earliest countries that adopted the United Nations Convention on the Rights of the Child (UNCRC). The different sectors in the country are therefore committed towards ensuring a safe and supportive environment to all children regardless of their socioeconomic background [1]. The services in the form of early education, child health services and social services are offered free of cost until the age of 18 years to ensure it is accessible to all children in the country. These services are focused on not only harm prevention but also to promote overall wellbeing [2–5]. However, despite these efforts, not all children in Sweden experience these rights equally [6]. According to a report by Save the Children, child poverty is concentrated in urban neighborhoods in larger cities in Sweden such as some areas in the city of Malmö which are regarded as socioeconomically disadvantaged or vulnerable [7]. These neighborhoods are characterized by high rates of unemployment, overcrowded housing, limited access to leisure time activities and increased concerns for safety owing to high rates of criminality. A significant portion of families in these neighborhoods have diverse migration backgrounds and often experience challenges accessing services, education and social participation owing to language barriers. A combination of these social and economic challenges represents important social determinants of health that contribute to inequities in children’s opportunities for wellbeing [8, 9].

Persistent inequalities in child health and wellbeing raises critical questions concerning how the national policies have been adapted to serve the growing diversity driven by rapid migration in the Swedish society [10]. As emphasized by Marmot, a one-size fit all approach to child wellbeing fails to account for the social gradients in health influenced by determinants such as poverty, exclusion and neighborhood conditions [11]. These complexities have also been discussed in the Bronfenbrenner ecological systems theory [12] which highlights how the different layers within a social context interact to shape children’s development. According to this theory social surroundings affect how children particularly those from migrant backgrounds form a sense of identity, interact with others and access opportunities for personal and social development. These factors play a key role in children’s health and wellbeing with lasting effects extending into their adulthood [12]. Furthermore, previous research suggests that the neighborhood has a role in shaping children’s behavior, attitudes and values which may also influence their overall wellbeing [13, 14]. Therefore, the World Health Organization (WHO) recommends early investment in children’s wellbeing, with the aim of reducing health inequalities, improving educational outcomes and promoting societal cohesion [15]. Child wellbeing is a multidimensional construct that includes physical, emotional, social, and material aspects. Although several definitions exist, research argues that wellbeing is a subjective perception which is shaped by cultural, social, economic and other contextual factors. United Nations International Children’s Emergency Fund (UNICEF) defines overall wellbeing as a multidimensional state that includes children’s health, safety, education, relationships and subjective experiences, shaped by the environments and opportunities that support their development [16]. This also entails that the meaning and significance of child wellbeing may vary across communities particularly for children with migration backgrounds navigating multiple cultural contexts. Thus, recognizing child wellbeing as inherently relational is critical for developing frameworks that are responsive to diverse needs especially in contexts shaped by migration and socioeconomic disadvantage [17]. Therefore, without a deeper understanding of how children with migrant backgrounds from socioeconomically disadvantaged neighborhoods themselves experience and define wellbeing, policies and interventions to promote wellbeing may not be successful [18]. This also highlights the importance of engaging children themselves in the research process to adequately capture their lived realities.

The Article 12 of the UNCRC reinforces that children have the right to freely express their views and should be involved in all matters that concern them [19]. Although child rights are recognized and even discussed actively in Swedish schools and within social services [20, 21], children are often merely consulted not involved in decision-making processes making it a tokenistic participation [22]. Sheir in his work on child participation challenges the paternalistic view that assumes that children lack the capacity to participate and contribute on equal terms with adults [23]. He further argues that the assumption limits children’s influence especially in matters concerning them thereby challenging the possibility to fully realize child rights. Shier’s work also emphasizes that children in general have the potential to express their values and goals even at an early age but may need reaffirmation from adults [23]. While guidance from adults may be necessary particularly in ensuring safety, child participation is not just about passive listening [24]. This disconnect between recognizing children’s rights and actively including their voices highlights the need to rethink how traditional researchers and policy-makers view child participation [25–28].

In contrast to the paternalistic view towards child participation, Ballet, Biggeri, and Comim suggest a *capability approach* introduced by Amartya Sen. The *capability approach* views children as active participants in their communities acknowledging their own personal goals and unique ways of navigating in their environment. Viewing children from such a perspective lays foundation for a more inclusive and responsive form of inquiry which is centered on the lived experiences and perspectives of the children [28]. To move beyond tokenistic participation, there is a need for research that consider children as co-creators of knowledge [29]. One way to effectively operationalize the capability approach is Community-Based Participatory Research (CBPR). CBPR is a form of Action Research in organizations introduced by social psychologist Kurt Lewin [30]. CBPR is a collaborative approach where community members (including children) and academic researchers together with other locally relevant stakeholders work together in equal terms to define research goals, identify and implement methods and co-construct and analyze research results with an aim to improve health and social equity. CBPR emphasizes collaboration and shared decision making, allowing children to influence the process and outcomes of research [31]. This kind of engagement challenges traditional power dynamics and helps fill critical gaps in understanding through focusing on lived experiences of the children [29]. CBPR has successfully been applied in adult community contexts for over two decades [32]. Research suggests that its application with children requires particular methodological considerations to ensure that the childrens’ perspectives are authentically represented [33, 34]. Established CBPR studies have also shown that engaging children as co-researchers can improve the relevance of interventions, improve health outcomes and enhance community resilience by incorporating children’s lived experiences into the program design [35–37].

In neighborhoods like those in cities in Southern Sweden where children face unique social and environmental challenges [38], participatory research can provide insights that is integrated in their life circumstances [39]. Recent research suggests that, in order to mobilize genuine knowledge especially together with children in vulnerable contexts, there is a need to apply innovative participatory methods such as arts-based activities, storytelling, and play-based techniques. These approaches have been highlighted as important tools for ensuring that children’s voices can shape research processes in child health and wellbeing [40]. Such approaches have also been shown to be valuable in contexts marked by health inequities, where children’s perspectives can illuminate how structural conditions shape their health and wellbeing [34].

Studies exploring child wellbeing using participatory approaches exists predominantly in context outside of Sweden but most of these centers focus on adolescents or young adults, often within structured educational settings [41–43]. These studies prioritize school-related experiences or explore the impact of health promotional interventions on child wellbeing with little or no attention to the broader socioeconomic conditions that shape children’s daily lives.

In addition, the level of child participation in many of the existing studies remains limited as children are often merely consulted or invited to express views, but rarely involved in shaping or reshaping research questions, identifying suitable methods, interpreting findings or influencing decisions [44]. To the authors’ knowledge studies focusing on defining child wellbeing together with children are relatively dearth. This gap highlights the need for context-sensitive research that centers children’s voices, especially those with migrant backgrounds living in socioeconomically disadvantaged circumstances. Such knowledge is also essential for addressing inequities that are embedded in the social conditions that shape their lives.

*Thus*,* this study aims to explore through a participatory process how children with migrant backgrounds in socioeconomically disadvantaged neighborhoods define wellbeing.*

## Method

The current study is part of a larger program Kids in Action for Health Equity (KAHEQ) which focuses on wellbeing among children living in socio-economically disadvantaged neighborhoods in Malmo city located in Southern Sweden [45]. KAHEQ is a CBPR program where children, parents, peer activity leaders, and researchers are engaged in co-researching child wellbeing and co-developing a culturally aligned survey tool to assess the wellbeing of children living in similar context participating in leisure time activities. As the first step in this co-development process, the present study engages children as co-researchers in defining their own wellbeing so that the findings can be integrated into the ongoing development of the survey.

### Study design

**Fig. 1:**
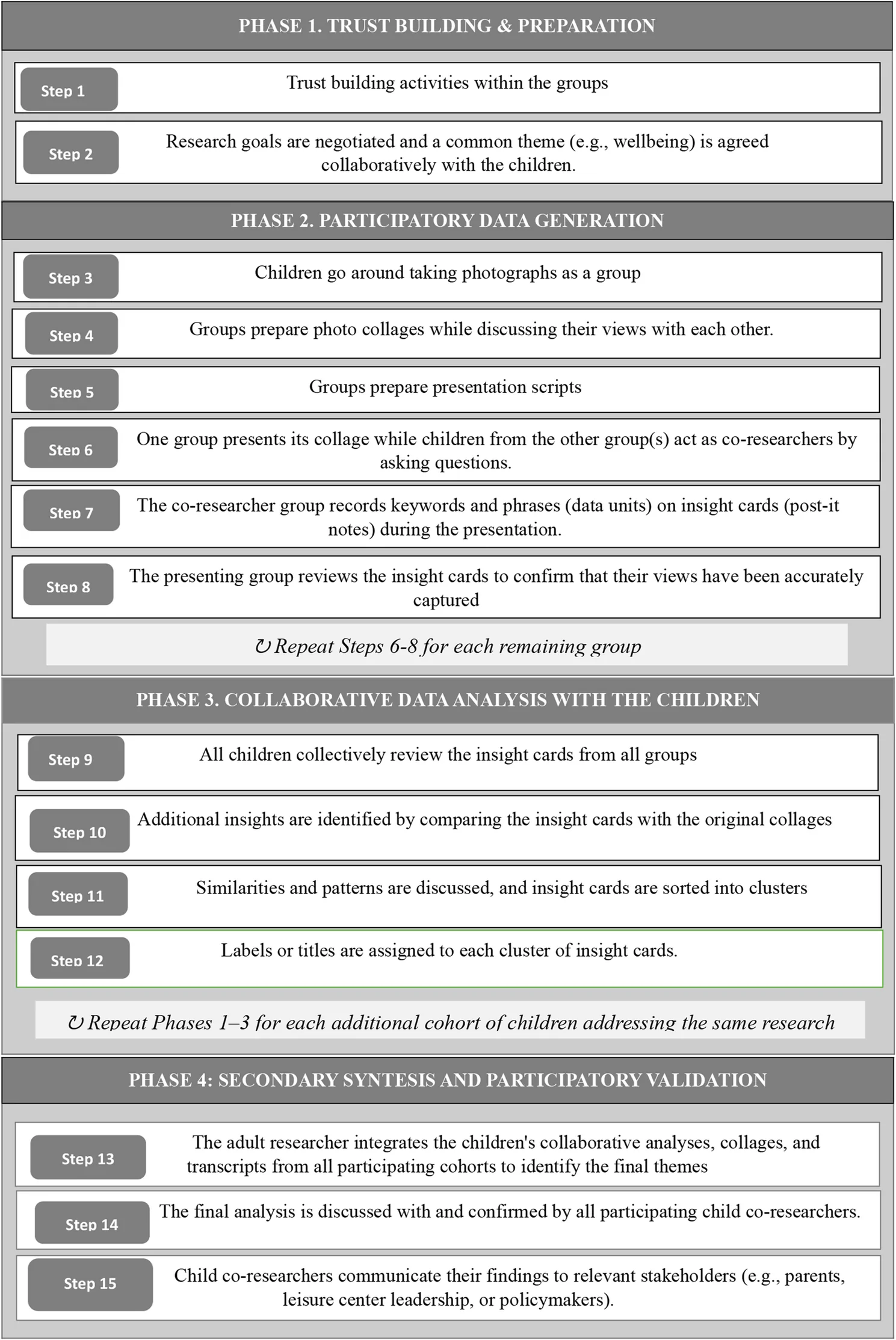
Step by step process within the Participant Led Collaborative Photovoice method

This study operationalizes the CBPR principles of the larger KAHEQ program [45] through a participatory action research approach involving children as co-researchers. Thus, children in this study were not merely consulted but were rather active as co-researchers in exploring how wellbeing is defined and experienced in their social context. A multistage focus groups design [46] was applied where the photovoice method [47] was used to facilitate discussions with children. The multi-stage focus group design was the overarching design within the KAHEQ program, planned by the researchers as participatory action research approach requires long-term engagement with the same group of children to build trust, continuity and shared decision-making. The multi-stage focus group design is a qualitative method in which a series of focus group meetings are conducted over an extended period where a range of activities and participatory methods are used to explore the research topic over time. The sessions are usually designed sequentially, with each meeting informed by and expanding upon the insights generated in the preceding session [46]. In this study, each of the weekly sessions held over a period of twelve months starting in June 2023- June 2024 was treated as a distinct focus group. During the initial phase, the children chose to use photographs as a starting point for their discussions, which led to the inclusion of the photovoice method in the current study. As the participatory process progressed and the children increasingly took ownership of the research activities, together with the researchers and the activity leaders they began reshaping the method by introducing more creative and collaborative elements. This gradual shift resulted in the development of a modified version of photovoice, termed the *Participant Led Collaborative Photovoice* (PLCP) method, which emerged organically from the children’s evolving engagement. Figure 1 illustrates the different steps within the PLCP method.

### Study context and selection of study sites

The children participating in this study were involved in activities organized by the All-activity Houses *(hereafter referred to as a leisure time center)* a social meeting place for children and young people in neighborhoods that are among those identified as socio-economically disadvantaged in Sweden. The *leisure time center* is an initiative by the Malmo Municipality located in the premises of schools in these neighborhoods. Although it operates within school buildings, it functions independently from the school system. Each *leisure time center* is managed by a manager, who is responsible for recruiting and supporting the activity leaders and coordinating with schools and local stakeholders. The *leisure time centers* are open throughout the week, including weekends, offering a wide range of free leisure-time activities including those involving families in the local community. These activities are facilitated by activity leaders and are developed in dialogue with the children themselves, ensuring that the content is relevant, engaging, and empowering for them. Activity leaders are key persons within the *leisure time center* who are chosen to reflect both gender and local diversity. The activity leaders had backgrounds similar to those of the participating children and were often born and raised in the same neighborhood or in other socioeconomically disadvantaged areas. Several of them had even attended the leisure center themselves when they were younger. They play a vital role in fostering trust, encouraging participation, and promoting inclusion among the children. Their responsibilities include organizing and leading activities, encouraging children’s engagement, and initiating conversations around important social and health-related topics.

The selection of study sites was based on the organizational status of the leisure time centers at the initiation of the KAHEQ program and their capacity to support the long-term participatory research process. At the initiation of the KAHEQ program, four leisure time centers were operating in socioeconomically disadvantaged neighborhoods in Malmö. Three centers located in Lindängen (Area 1), Rosengård (Area 2), and Helenholm (Area 3) were selected for inclusion in the study. The fourth center had only recently commenced activities and was operating in collaboration with the Lindängen center during the planning phase of the project. Therefore, it was not included as an independent study site. The selected centers provided geographical representation across different socioeconomically disadvantaged neighborhoods in Malmö while also offering established organizational structures capable of supporting the long-term participatory activities required for the study.

### Participants

The study involved 31 children (15 boys and 16 girls) aged 10–12 years from the three participating leisure time centers, corresponding to approximately 10 children per site. All participants had migrant backgrounds and were predominantly from families originating from the Middle East, while some were from Eastern European countries. All participating children were required to understand and speak Swedish. This age group was selected because evidence suggests that children from approximately 10 years of age possess the verbal, cognitive, and social abilities needed to actively participate in collaborative discussions and co-research activities. Approximately 10 children were included from each site because the study was designed as a long-term Community-Based Participatory Research process involving weekly meetings over a one-year period. The methodology relied on extensive trust building, collaborative decision making, and children’s active involvement as co-researchers throughout both data generation and analysis. A group size of approximately 10 children per site was considered sufficiently large to generate a diversity of perspectives while remaining small enough to facilitate meaningful participation, relationship building, shared decision making, and active engagement of all children throughout the extended participatory process. Establishing one co-research group within each leisure time center also enabled representation from multiple disadvantaged neighborhoods while maintaining the continuity and intensive engagement required for the participatory research approach.

### Recruitment

The leisure time centers introduced a *Research Group* activity as part of their regular activity program and invited interested children to register with parental consent. The researchers did not participate in selecting participants but conducted informational meetings in each area to present the larger research program. Written information about the study was subsequently distributed to the parents of children who expressed an interest in participating.

Children were enrolled in the order of registration while aiming to achieve a balanced representation regarding age and gender. Participation was voluntary, and both parents and children were invited to contact the leisure time center to express their interest in participating in the study. All children enrolled in the activity and who provided informed consent, together with parental consent, were included in the study. Following the first few meetings, the children decided to rename the activity “*Active Kids*”.

**Fig. 2:**
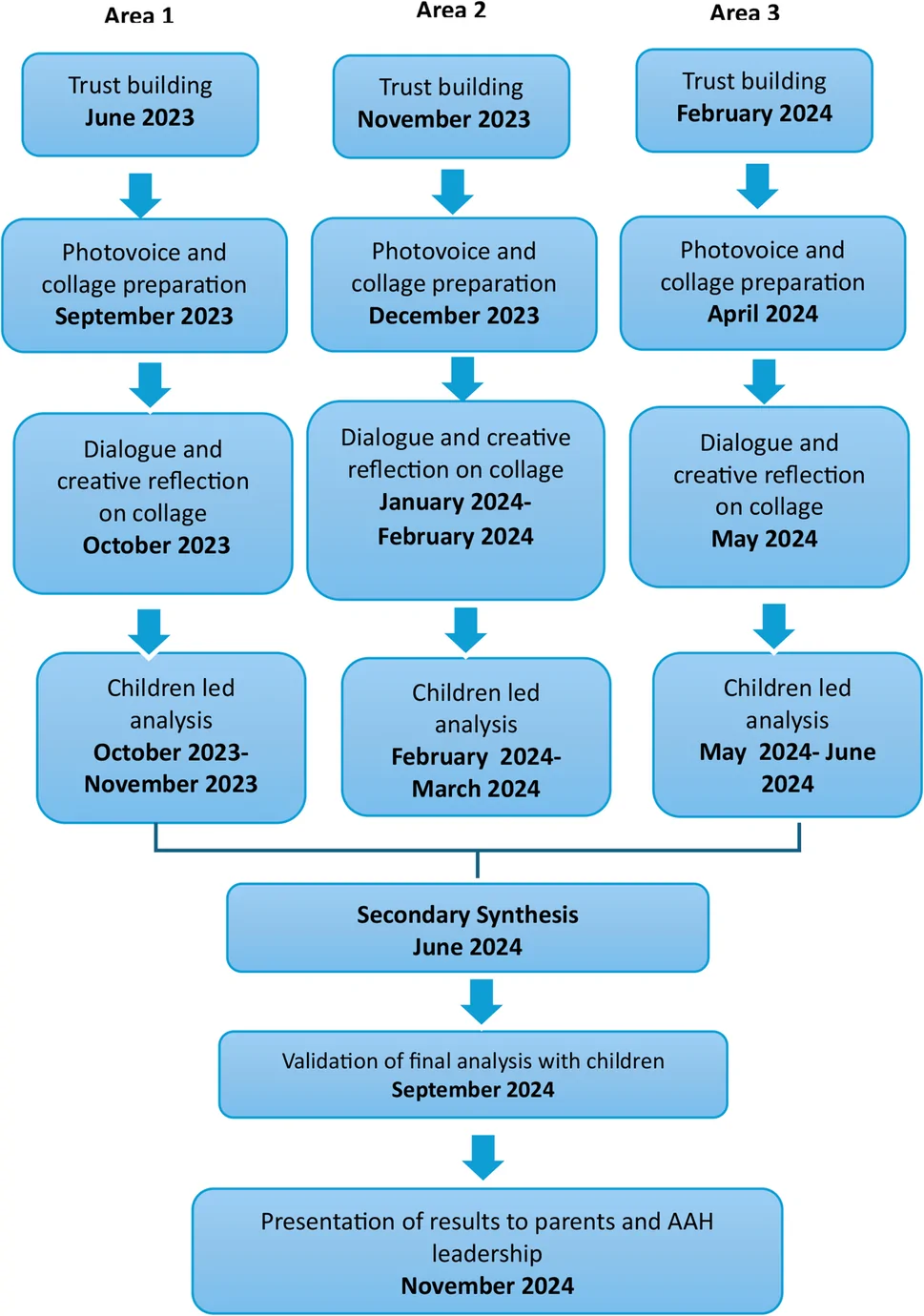
An illustration of the participatory timeline in the three neighborhoods

Although all the children were interested and attended the *Active Kids* activity regularly, the number of participants in each weekly session varied. Some missed sessions due to schedule clashes with mother tongue classes or homework support sessions, medical appointments, or illness. However, the multistage focus group design itself allows changing participant dynamics without disrupting the discussions [46]. In addition, each session began with a brief recap of the previous week’s activities, during which the children actively reminded one another of what had been discussed. They also took the initiative to explain key points to those who had missed the earlier session, helping them rejoin the conversation and stay engaged with the ongoing discussion. Figure 2 presents an illustration of the participatory timeline in the three neighborhoods.

### Community-based participatory research team

A Community-Based Participatory Research (CBPR) team was formed in each leisure time center, consisting of the participating children, the first author, and a PhD student. The activity leaders acted as bridges between the research team and the children. A total of five activity leaders participated in the CBPR teams. In Area 2 and Area 3, one male and one female activity leader participated, while Area 1 included one female activity leader. The CBPR teams also included senior researchers from the larger project who were not directly involved in the field activities but had advisory roles and contributed through discussions during bi-weekly research meetings. Each *Active Kids* session lasted approximately two hours and always began with an evening snack provided by the leisure time center, consistent with the center’s regular activities. This meal was considered important as many children arrived after a long school day and were often tired and hungry. Following the meal, the group engaged in research-related activities. The sessions concluded with what the children called a “surprise snack”, which they regarded as a form of appreciation for their contributions as co-researchers.

### Trust building phase

As in any CBPR study, trust building was considered crucial for fostering a safe environment in which children feel respected and empowered to share their perceptions openly and confidently. Previous research suggests that partnerships grounded in trust can reduce power imbalances, enhance the quality of discussions, and promote sustained engagement among co-researchers [48, 49]. The trust building phase began even prior to the establishment of *Active Kids* activity and it was the very first step in this study. The first author visited the *leisure time center* regularly, participating in various activities to become familiar with the organization and its personnel, in particular the activity leaders, while also ensuring a visible presence among the children. Establishing a partnership with the activity leaders further enhanced opportunities to engage with the children, as these leaders were already trusted figures in their lives. Following the establishment of *Active Kids*, the first author and a PhD student met weekly with the children and activity leaders in the three-leisure *time* center. A unique aspect of *Active Kids* was that, unlike other activities organized by the *leisure time centers* where children of the same age participated, this group included children of different ages and from different classes. Thus, the early phase of this study focused on at first building trust both among the children followed by between the children and the research team, with the activity leaders serving as key mediators in this process. The children were divided into two small groups to engage in team building activities where the research team also participated. This was followed by discussions on decision making and the value of children’s perspectives. The children were subsequently introduced to their roles as co-researchers. The children expressed a desire to have identity cards similar to those worn by the researchers, with their names and the words “Child Co-Researcher.” This was understood as a way for the children to affirm, both to themselves and to others, their role as active co-researchers in the project. They also visited the university to become familiar with the adult researchers’ work environment, further reinforcing their sense of inclusion and partnership in the research process. A common practice at *leisure time center* is that all children follow a set of rules for how to behave within the different *leisure time centers*. Since the children saw themselves as co-researchers, they chose to write the rules themselves instead of using ones made by adults. They created basic guidelines (code of conduct) about how to treat one another and how to respond in different situations, agreeing on them together. Alongside the rules, they also listed possible consequences for not following them. These consequences were gentler than those typically used in schools, as the children felt that strict punishments did not help them learn or grow.

Although the study began with a trust-building phase, it must be noted that trust was understood as a dynamic process rather than something established once and assumed to remain stable throughout entire time of this study. During the *Active Kids* sessions interactions were continuously negotiated together with the children through dialogue and reflection to identify and address any power imbalances that might arise over time. Each session began and ended with open reflections in which children were invited to talk about the group dynamics, any discomfort they had experienced, whether they felt that adults or other children had taken too much space, and whether they felt unheard. Issues that emerged in these reflections were also followed-up in the weekly planning meetings with the activity leaders to ensure that concerns identified by the children were addressed. These reflections enabled the activity leaders and the first author to adjust the facilitation of the sessions when needed. The *Active Kids* sessions involved joint decision-making through democratic voting where children and adults participated on equal terms. It was also ensured that adults did not outnumber the children in any session so that adult voices did not dominate decision-making. These practices were regarded as important because trust can weaken during a long participatory process, and children may feel less secure if they sense unequal treatment or if adults unintentionally take over.

### Data collection

The discussions in the first multistage focus group session [46] following the initial trust building phase began by exploring the meaning of the word wellbeing from the children’s perspective, and then gradually moved toward identifying ways to define wellbeing collectively *(Step 2).*

In collaboration with the research team, the children in the CBPR teams decided to photograph aspects of their lives that they associated with wellbeing. In each *leisure time center* the CBPR team was divided into groups, where each group was composed of 5–6 children supported by one activity leader together with either the first author or the PhD student *(Step 3).* The groups moved around and took pictures of objects, places, and people they perceived as connected to their wellbeing. Subsequently the children expressed a desire to make their work more creative by creating collages from the photographs they had collected. They also wanted color markers, pens and stickers to enhance their presentation. In the following sessions, the children worked on their respective collages, discussing within their groups how the collected photographs related to their well-being *(Step 4).* The discussions within the groups while working on the collages were audio-taped.

As a next step each group collectively prepared presentation scripts based on their group discussions related to their collages and later presented their collage to the CBPR team *(Step 5)*. The collages were thus created and presented as a group unlike in a traditional photovoice study where individual images are discussed [47]. Each *leisure time center* consisted of two groups *(Step 6)*. While one group presented, the other group who were audience acted as co-researchers, posing follow-up or probing questions to the presenting group as well as taking memos and creating small insight cards on post-it notes summarizing keywords or phrases from the presentation and discussion that were related to wellbeing *(Step 7)*. Unlike in a conventional photovoice method [47], where the adult researcher typically facilitates and probes participants’ discussions, in this methodology the children acted as co-researchers by questioning one another, eliciting deeper reflections, and collaboratively generating the analytical data. It must be noted that since the photovoice method relied on the photos chosen by the children and the evolving dialogue, a structured interview guide was not used. The discussions were instead guided by the children’s own questions to one another, such as why they chose a particular photo and what it meant for their wellbeing. The insight cards were then placed on a whiteboard in such a way that the presenting group could also correct or refine the insight cards to ensure that the interpretations remained child-led *(Step 8)*. The process was then repeated when the groups switched roles and the audience group presented their own thoughts on their collage and the first group acted as the audience, organizing the same co-researcher process and creating a new set of insight cards This process was repeated in all three *leisure time center*s with the respective CBPR teams. All sessions were audio recorded with the informed consent of both the children and their parents to enable detailed analysis of the group dialogues.

### Data analysis

In the following focus group session, the children reviewed the insight cards generated in their respective *leisure time center (Step 9)*, to add any additional insights that may have been missed while also referring to the collages *(Step 10)*. They then worked collaboratively to identify similarities across the data units. A participatory co-analysis process using insight cards was performed where children identified insight cards with related data units and placed them together in clusters *(Step 11)*. As a next step they discussed and agreed on labels (a title) that captured the essence of each cluster discussing why this was related to their wellbeing *(Step 12)*. This process led to the identification of overarching themes that represented the related data units. The discussions during this stage allowed the children to explain their reasoning and to negotiate differences in their interpretations. The collages, the children’s verbal explanations captured through audio recordings, the insight cards and the children’s preliminary analysis together formed the photovoice data. Following this participatory analysis, the first author carried out a second level synthesis *(Step 13)*. The data were examined in relation to the themes identified by the children together across the three *leisure time centers* together with verbatim transcripts of all the audio recordings while also reflecting simultaneously on the visual material namely the collages.

The process resulted in a refined and consolidated set of themes and subthemes that integrated insights from the children in all three *leisure time centers*. The final set of themes and subthemes were presented, discussed and confirmed within the CBPR teams together with the children *(Step 14)*. The children also took ownership of the analysis and presented the themes to their parents and *leisure time centers* leadership in the subsequent sessions *(Step 15)*.

### Ethical considerations

This study was part of a larger program approved by the Swedish Ethical Review Authority, also known as the Etikprövningsmyndigheten (DNR 2023-00979-01). The study was conducted in accordance with the Declaration of Helsinki, and participation was voluntary. Since the children who participated in this study were between 10 and 12 years old, their parents or legal guardians received written information explaining the study and its purpose and provided written informed consent for their child to participate ahead of the study. The parents were also informed about their children’s role in the program, including where the results would be presented and that participation would be anonymous so that no individual child could be identified. Written informed consent was also obtained from the children after providing verbal and written information about the study. Although formal consent was obtained in the beginning of the study, given the long-term design, ongoing reflections with the children were facilitated to ensure that their comfort, autonomy, and voluntary participation were maintained over time. The activities were conducted on the school premises, allowing the children to participate in a familiar setting and making it more practically accessible to them. Although the presence of the activity leader may pose ethical questions, they did not influence the children’s ideas or thoughts. Furthermore, the activity leaders supported the research team and the children if any sensitive discussions emerged in the group that made the children uncomfortable.

## Findings

**Fig. 3:**
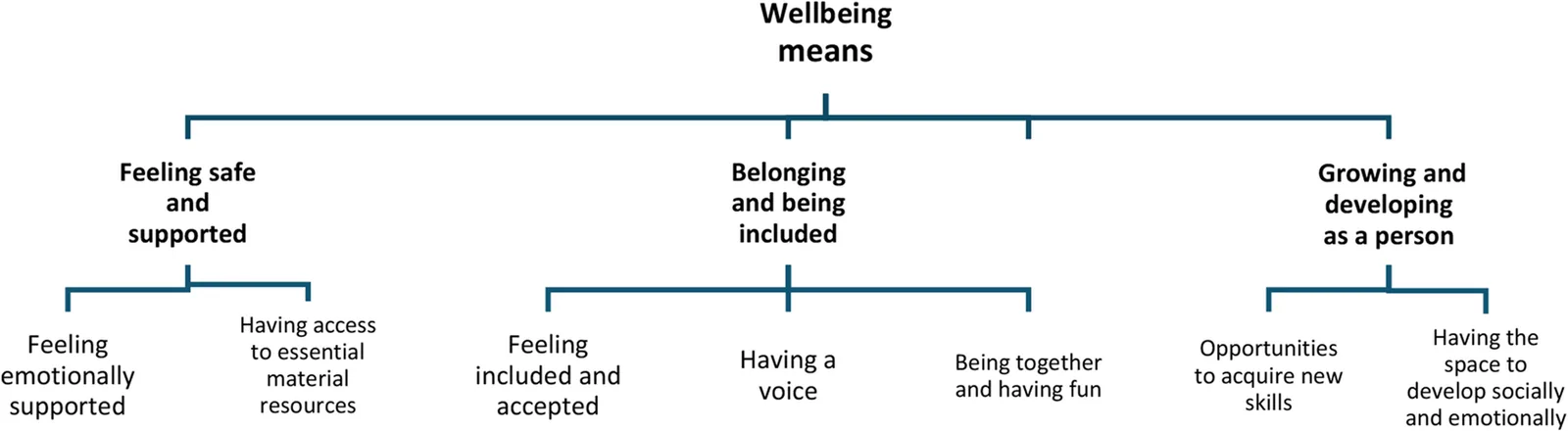
Conceptual structure illustrating children’s definition of wellbeing

Three core themes emerged from the discussions with the children: *feeling safe and supported*,* belonging and being included*,* and growing and developing as a person*. Each theme captured different yet interconnected dimensions of wellbeing, based entirely on the children’s own voices and values. The core themes and subthemes are presented in Fig. 3.

### Feeling safe and supported

Sense of security was discussed as a central aspect across the children’s reflections. They described the need to feel emotionally, physically, and socially safe as essential to their wellbeing. Security was viewed as dynamic, developing through their physical interactions, respectful communication with emotionally available adults, and the assurance of access to resources, including materials and a clean, calm living environment. Two subthemes emerged from this theme including *feeling emotionally supported* and *having access to essential material resources.*

#### Feeling emotionally supported

Children described feeling secure and experiencing a sense of wellbeing when they had adults they could approach for support. They also insisted that they felt comfortable with adults who were willing to listen, in contrast to those who spoke harshly. The children in this study preferred adults who engaged in dialogue rather than those who were judgmental when conflicts emerged.

*“You feel safe knowing that you have an adult to talk to…And that makes us feel good.” (Girl*,* 10 years*,* Area 1****)***.

The children also highlighted that raising voices was not seen as a means to discipline, but as something that made children feel emotionally unsafe as it pushed them into an uncomfortable state and affected their wellbeing.

*“If adults yell*,* it doesn’t feel good. It feels better when you get to talk about things instead of just being yelled at.” (Girl*,* 11 years*,* Area 3)*.

#### Having access to essential material resources

The children believed that their state of wellbeing was defined by access to essential resources, including surplus food, a clean environment, and equipment that supported sports and play during leisure time. They described these resources are not privileged, but rather fundamental to feeling healthy, active, and socially connected.

Children described their immediate physical environment as vital in shaping their sense of wellbeing. They emphasized that a clean, organized, and pleasant living environment was important for feeling well. The presence of dustbins in public spaces and around schools, along with access to clean toilets, was identified as contributing to their wellbeing.

*“It’s good when it’s clean around you*,* and then you feel better. When the toilet smells nice… it feels safer and you feel good.” (Girl*,* 10 years*,* Area 1)*.

The children stated that food was essential not only to satisfy their hunger but also to provide the energy needed to manage their day. They further emphasized that it was not about merely having access to food but that there was enough that it allows them to eat freely without restrictions. The children related having surplus of food to giving them a feeling of security and comfort.

*“We need to eat breakfast so that we’re not hungry and have enough energy. Food gives us energy. But we need to be able to eat as much as we want. Then we feel safe and feel good.” (Boy*,* 10 years*,* Area 2)*.

The children also highlighted the significance of having access to material resources that support their leisure activities, recognizing these as essential for their well-being. They expressed that engaging in play and sports contributed to how they felt physically and emotionally. The children further highlighted that for their leisure time to be truly fulfilling, it was important not only to have opportunities for such activities but also to have adequate sports equipment and play materials. The children believed that access to these sports equipment enabled them to fully participate, enjoy, and benefit from their leisure time, reinforcing their sense of wellbeing.

*“We need things to play with so we can have fun during our free time and recess. Playing football is important because it helps us feel happy and feel good. If we don’t have balls and other things*,* it becomes boring and we do not feel good.” (Boy*,* 12 years*,* Area 3)*.

### Belonging and being included

The discussions with children in this study showed that wellbeing was closely connected to the sense of belonging. Children discussed that being part of social circumstances and being accepted, included, and being able to express themselves as vital to their wellbeing. Two subthemes emerged from this theme including *feeling included and accepted*, as well as *having a voice.*

#### Feeling included and accepted

The children emphasized the importance of fairness and diversity and stated that being treated with respect regardless of their gender, economic background, language, appearance, or religious beliefs influenced their wellbeing. Many children expressed the importance of equality, stating that it should be considered as a right rather than as a privilege.

*“Everyone is equally valuable*,* boys and girls. It doesn’t matter what religion you have or what you look like. You feel good when you don’t feel judged*,* even if you’re different.” (Girl*,* 10 years*,* Area 2)*.

#### Being together and having fun

Children described happiness as emerging from social connection, joy, and shared experiences, all closely tied to their wellbeing. Happiness was not seen as an individual experience, but as a collective journey, expressed through relational joy through being together and engaging in activities with others. They discussed shared emotional experiences perceived when spending time with friends, especially during group activities such as games, dancing, drawing, music, or simply being together. Being with friends made them happy, as they would otherwise feel bored and alone at home

*“You feel happy just seeing your friends*,* and we laugh a lot when we’re together… you don’t feel alone anymore*,* and you feel better.” (Boy*,* 10 years*,* Area 1)*.

#### Having a voice

The children also discussed the importance of being heard and getting involved in decision making regarding matters that concern them. Children emphasized how having influence and being allowed to make decisions, such as democratic decision making through voting and polling to offer ideas made them feel important. The children said that their wellbeing was linked to participation, which is not superficial but rather emotional as they started to see themselves as important when their ideas were considered seriously.

*“We get to be part of making decisions… if you say something and it is taken seriously*,* it means a lot*,* then you feel important.” (Boy*,* 11 years*,* Area 3)*.

### Growing and developing as a person

Children spoke about wellbeing not only as an emotional feeling but also as something related to how they were evolving as a person. They associate growth with learning new things, developing skills, and gaining confidence especially through playful, creative, and active experiences. This theme included two subthemes, namely, *opportunities to acquire new skills* and *having a space to develop socially and emotionally*.

#### Opportunities to acquire new skills

Children described the process of learning beyond school education, through participating in leisure activities such as playing games, engaging in sports especially as a team, and practicing art. The children explained that engaging in dance and other forms of body movement gave them positive energy, helped relieve stress, and contributed to their overall wellbeing. They emphasized that constructive use of their leisure time provided opportunities to explore, experiment, and develop their skills in sports and creative expressions, including drawing, painting, and crafting, as well as performing arts such as dance and music.

*“It is important that we get to try new things that we have never done before*,* you grow. Things we usually do not get to do like learning to ride a horse.” (Girl*,* 10 years*,* Area 2)*.

Children described experiencing a sense of having learnt and achieved when they had access to try new things or developed interests in things they had not previously explored. The act of thinking independently, being creative, and being able to choose contributed not only to their learning but also strengthened their self-confidence.

*“When you get to think for yourself*,* come up with ideas*,* and do different things*,* it makes you feel smart*,* you can feel yourself growing*,* and it makes you feel good.” (Girl*,* 10 years*,* Area 3)*.

#### Having the space to develop socially and emotionally

Children also described development and learning to their emotional growth which in turn was related to their overall well-being. They explained that participating in activities helped them improve their communication skills, become more empathetic, and build stronger social relationships, all of which made them feel well in their social environment.

*“It is not just about having fun*,* but also about becoming a better person. We have learned how to behave and how to be a good friend*,* that is what is important for us to feel well” (Boy*,* 12 years*,* Area 2)*.

## Discussion

The results of this study suggest that children with migrant backgrounds living in socioeconomically disadvantaged areas describe wellbeing in terms of feeling safe and supported, belonging and inclusion, as well as having opportunities for growth and development. These perspectives show that wellbeing emerged from relationships, material conditions, and opportunities available in their immediate environments to learn and play. It is also worth noting that the children emphasized basic material needs such as food, clean toilets and access to equipment, since these are usually assumed to be guaranteed in a welfare‑state context where all children are expected to have equal access to such resources [4]. These findings can be interpreted through the lens of Bronfenbrenner’s ecological systems theory [12], which highlights how children’s wellbeing is shaped through dynamic interactions between relational, material, and structural contexts. At the microsystem level, children emphasized the importance of calm, responsive adults and inclusive peer relationships. At the exosystem level, access to material resources such as food and equipment shaped their sense of security and participation. This may also reflect the reality that many migrant families in socioeconomically disadvantaged neighborhoods may have limited financial resources, making access to food and sports equipment particularly important for children. At the macrosystem level, children articulated values of fairness, equality, and rights, reflecting broader societal norms that influence their daily experiences. The mesosystem did not directly appear in the children’s accounts, because the children defined wellbeing through what they directly experienced rather than through connections between different contexts such as home and school.

The children’s definition of wellbeing in this study also align with UNICEF’s definition of wellbeing [16] yet the children’s accounts extends this definition by highlighting contextual experiences shaped by material and relational conditions that influence their sense of safety. The children’s emphasis on surplus food, clean toilets and access to equipment demonstrates that children themselves conceptualize basic material conditions as foundational to feeling safe, valued, and able to participate, an aspect that previous research has not captured from children’s own perspectives. For children with migration backgrounds, such material conditions often overlap with challenges like financial strain, crowded housing, or limited availability of supportive community resources [50]. The children’s focus on basic material needs may also indicate an awareness of poverty-related constraints in their life circumstances, where limited household resources make access to food and equipment uncertain. However, previous research has shown that reliable access to essential resources reduces stress and supports emotional and cognitive engagement, with food security and access to sport equipment specifically linked to improved health outcomes [51, 52]. Children in this study described feeling safe when they were in the presence of adults who were calm, attentive and available to them. They described calm, predictable communication from adults as essential for feeling understood and safe. These findings are also in line with attachment theory, which highlights how emotionally available and responsive adults provide the secure base that supports children’s emotional wellbeing and enables them to explore their environment with confidence [53]. Furthermore, children’s sensitivity to tones, specifically the preference for calm communication over raised voices, points to the developmental importance of interactional communication climate for feeling safe. This resonates with prior evidence that when parents or other adults in children’s life such as teachers and other school staff in authoritative positions behave harshly or strictly, it increases the risk of mental ill-health among children [54]. While previous studies emphasize caring-adult support, few capture children’s explicit distinction between calm, predictable communication and raised voices as direct determinants of child wellbeing. Similar patterns have been shown in another study from two multicultural neighborhoods in Sweden, where *leisure time centers* were experienced as health-promoting environments because activity leaders provided calm, supportive and predictable adult interactions [55].

Children in this study also described wellbeing as closely connected to belonging and inclusion. They described fairness, equality, and being treated with respect regardless of gender, religion, language, appearance, or socioeconomic background as rights that shaped their wellbeing. These reflections may be pronounced in these children given their migration backgrounds, as they often navigate multiple cultural expectations and may be more exposed to exclusion or discrimination. Their emphasis on equality and fairness may thus reflect their lived experience of lacking certain resources, making them more aware of issues of inclusion and rights. This resonates with prior research demonstrating that social inclusion and positive peer relationships are central to young people’s lived and social experiences, shaping perceptions of acceptance and integration in the society [56]. Further, research among Swedish adolescents also show that exclusion related to migration background, religion, ethnicity, or visible difference undermines adolescents’ sense of belonging [57]. Although previous studies have shown that social inclusions support children’s social experiences, they have not shown how children themselves define inclusion, fairness, equality, and respectful treatment as a core component of wellbeing rather than simply a social experience. The findings from this study therefore contribute new insights by demonstrating that children conceptualize inclusion not only as a social condition but as a fundamental determinant of their wellbeing.

Another central part of belonging for the children in this study was having a voice. They described participation as emotionally meaningful rather than superficial, explaining that being listened to and having influence in decisions that concerned them made them feel important. This finding could be linked to the Article 12 of the UN Convention on the Rights of the Child [58], which guarantees children the right to be heard. Children in this study even distinguished between tokenistic involvement and genuine participation that affirmed their competence and autonomy, an aspect that is rarely captured in existing research. This finding aligns with prior evidence showing that children’s participation in daily life is positively associated with their subjective wellbeing, particularly when participation involves meaningful influence rather than symbolic inclusion [59]. While participation is widely discussed as a policy and also ratified in countries like Sweden [20], studies focusing on children’s own differentiation between symbolic involvement and meaningful influence, or how genuine participation contributes directly to their wellbeing are distinct. Previous research also indicates that for children with migration backgrounds, meaningful participation may help mitigate experiences of marginalization or restricted agency encountered in other settings [60].

Children in this study described wellbeing as emerging from growing and developing as a person. Happiness was described as a collective experience rooted in social connection, shared activities, and relational joy, which is consistent with prior research showing that children often define wellbeing through friendships, shared play, and feeling connected to others [61, 62]. These descriptions also resonate with Vygotsky’s sociocultural theory [63], which emphasizes that development is shaped through interaction, collaboration and shared experiences. This connection is especially relevant in participatory action research as in the context of this study, where children’s playful interactions and shared problem solving are treated as meaningful forms of knowledge that reveal how they understand and shape their own development. Vygotsky argued that play creates a unique space where children can take on new roles, experiment with ideas and act beyond their usual level of competence, which makes these playful interactions particularly valuable for understanding how children learn and develop [63]. Activities such as games, dancing, drawing, and spending time with friends provide emotional nourishment and support children’s autonomy and competence. Children in this study also described wellbeing as growth through learning new things, developing skills, and gaining confidence in playful, creative, and active experiences. They emphasized learning beyond school through sports, arts, and movement, which offered opportunities to explore, experiment, and build mastery. This aligns with evidence that participation in their routine activities and opportunities to develop competence are linked to higher subjective wellbeing [59, 61]. However, previous studies have not shown how children themselves conceptualize growth, learning, and the development of confidence as an intrinsic part of wellbeing rather than simply a factor that enhances it. The current study instead demonstrates that children with migrant background living in disadvantaged neighborhoods view these opportunities not only as enjoyable but as essential for feeling capable, confident, and developing as a person. This may also reflect that limited financial resources at home make community-based activities such as the *leisure time center a* particularly important arena for development, as they offer opportunities children might otherwise lack. This also aligns with another study from multicultural neighborhoods in Sweden showing that leisure-time youth centers were described as important environments for developing confidence, skills and a sense of capability among young people [55].

In the current study, the aim was to understand wellbeing from the perspective of children with migration background residing in disadvantaged areas. However, it was not possible to compare their experiences with those of children with a Swedish background. Conducting such a comparison in future research would be valuable, as it could provide deeper insight into potential differences and further illuminate how wellbeing is shaped across.

## Conclusion

The study shows that the understanding of wellbeing among children with migrant backgrounds is embedded in the immediate environments that shape their lives. As wellbeing has been increasingly recognized as a key social determinant of health, these findings also highlight how inequitable conditions shape children’s opportunities for healthy development. Their descriptions of safety reflect emotional and physical wellbeing, and their accounts of fairness, inclusion, and being listened to reflect social wellbeing. Their emphasis on opportunities to explore, play, move, create, and develop skills reflects cognitive and physical wellbeing, supported through access to experiences that are not consistently available in their settings and are often underrepresented in existing research. Several aspects the children identify as central to their wellbeing are often assumed to be guaranteed in settings with more secure material and relational conditions and therefore may not appear as defining elements in studies assessing child wellbeing. These findings further highlight how structural disadvantages may hinder migrant-background children’s access to environments that support health and wellbeing. Furthermore, their perspectives on wellbeing were also shaped by having a migration background as it relates to the social and material conditions of these neighborhoods, influencing feelings of belonging, identity, and access to supportive environments. This contrast illustrates how children’s understandings of wellbeing are shaped by the conditions they navigate and why their perspectives must be interpreted in relation to the environments that frame their lived realities. To sum up, these findings leans towards important health equity implications, emphasizing the need for community settings that can compensate for inequitable access to resources that support and promote child wellbeing.

## Strengths and limitations

An important strength of this study is the level of children’s participation, especially their direct involvement in analyzing the photovoice material and discussion notes. The children grouped insight cards, compared interpretations, and identified themes together. It must also be noted that the insight card step and the co-analysis process were not part of the original photovoice method [47] and were developed together with the children in this study, highlighting how the children shaped and expanded the methodological process rather than simply following it. The children’s active role in analyzing the photovoice material reflects higher levels of participation within Shier’s Pathways [23], where children influence the research process rather than simply contribute data. Shier argues that when children move beyond simply giving views and begin to take part in analysis and decision making, they enter the higher levels of the participatory pathway, where power is shared and children influence the outcomes of the process. This form of participation is seen as both more ethical and more meaningful, because it treats children as competent social actors whose interpretations shape the knowledge that is produced [23]. Another strength of the current study was that the overall procedure was age appropriate in that it was adapted to the capabilities of children aged 10 years and above. Firstly, the study was grounded in a participatory action research approach, whose core principles include adapting to children’s capacities, with consideration to age and background, because it emphasizes active involvement, shared decision making and flexible modes of expression [29, 64]. In this study the process was agreed at each step together with the children, who completed all stages of the study and engaged with each activity as intended. The children were also encouraged to indicate any challenges during the process, allowing the steps to be adapted to their needs as the research proceeded. Secondly, the photovoice method has been frequently applied with children aged 9–12 years in prior research which demonstrates that the method is well suited with children’s age and cognitive ability to communicate their experiences [37, 65, 66]. Wang and Burris also suggest photovoice as suitable method for younger children, especially those from marginalized groups, because the method involves producing and discussing photographs, activities that do not rely on high levels of literacy and allow participants to express their views in ways that are suitable to them [47]. Despite being age-appropriate a potential limitation in this photovoice study could be that children may have only photographed material objects that symbolize or relate to their wellbeing. However, in this study material objects were only symbolic, as children’s reflections moved much beyond them into emotional, social and cognitive aspects of wellbeing as shown in the results. In addition, when the children presenting their collages responded superficially to certain images, other children asked probing questions and discussed the pictures further, which deepened the reflections and helped shift the focus away from the material object. According to Wang and Burris the photographs function only as starting point and that the meaning develops through group discussion [47]. They also explain that participants often photograph concrete objects, but deeper interpretation emerges through dialogue, which helps move the discussion beyond the object, as was done in this study.

Another possible aspect that may be regarded as a limitation in this study was that participation was not consistent across sessions as some children were occasionally absent due to illness or other commitments such as extra coaching or mother tongue lessons. This project used a multistage focus group design, where variation in group participation from session to session is inherent owing to the longitudinal nature of the design [46]. In this study, these changes were addressed by beginning each session with a recap given by the children, which helped maintain continuity and ensured that all child co-researchers could follow the ongoing activities. One other limitation, consistent with participatory action research studies, is that the findings reflect the perspectives of the children who took part in this specific CBPR program. However, this study did not aim to capture all possible experiences of children with migration backgrounds but instead offers situated insights into how children in these neighborhoods understand wellbeing. The presence of activity leaders during the sessions may be regarded as a potential limitation in this study, as it could have influenced the extent to which children felt able to express their views openly. However, the activity leaders within the CBPR team had an essential role in facilitating trust between the researchers and the children, supporting communication, and thus acted as relational and cultural bridges. They also took part in weekly planning meetings together with the first author, where power differences were discussed and participatory values were reinforced alongside practical planning for upcoming *Active Kids* sessions. In these weekly planning meetings, continuous reflection took place regarding the power imbalances in the groups, including how the activity leaders’ broader interactions with the children outside the *Active Kids* sessions could both support and potentially influence the research process. Although, the activity leaders met the children in other activities organised by the leisure-time center, they never met them in the same group compositions as in the *Active Kids* sessions. The activity leaders were informed to bring back any concerns raised outside or during the sessions to the weekly meeting for discussion before formally addressing it, as this was one of the agreements established within the CBPR team. The children were also informed that discussions within the *Active Kids* sessions should remain within the group, meaning that any issues needed to be raised with the activity leaders and the first author before or after the *Active Kids* session. It was noted that there were many instances where the children spontaneously shared positive experiences about being part of *Active Kids* rather than raising problems, and the activity leaders reported these moments back to the research team. In addition, the activity leaders were trained as part of their employment process in the leisure-time center in ways that prevented them from exerting power over the children or influencing the children’s thoughts, as the center’s foundational values emphasise that children and adults have equal voices and must be allowed to express themselves in ways they find comfortable. Through these measures the research team aimed to ensure that the interactions of the activity leaders with the children did not influence the children’s views in a way that would compromise the study’s findings but rather enabled them to safely participate in the discussions. The role of the activity leaders in this study can be compared to the function of cultural brokers described in prior research [67, 68], as they facilitate communication and understanding across relational, linguistic, and contextual boundaries to promote more equitable participation rather than hindering it.

### Practical implications of the participant-led collaborative photovoice method

The Participant Led Collaborative Photovoice Method has several practical implications for future research using visual methods with children and for studies aiming to ensure meaningful participation throughout the research process. Drawing on the established Photovoice tradition [47], the method supports ethical engagement, and it also views children through the lens of the capability approach [28] as capable and competent, with inherent potential to contribute as equal partners in research. Grounded in Paulo Freire’s emphasis on dialogue [69], the PLCP creates structured opportunities for children to express, examine and refine their understandings of their immediate environments through sustained engagement with visual material. The method follows the action research process of think-reflect-act, and this structure is evident in the PLCP process where the participatory data generation in Phase 2 aligns with the think phase, the collaborative data analysis and participatory validation in Phase 3 align with the reflect phase, and the presentation of findings to stakeholders in Phase 4 aligns with the act phase. Similar to the traditional Photovoice method [47], the PLCP can be adjusted to the specific context in which it is carried out, and this flexibility allows the process to be shaped by the setting, the children involved and the practical conditions of the study. Furthermore, the method requires sustained engagement with the children over time, which makes it easier to implement in settings where children are already part of established routines, such as schools, youth centers and other organizations, while still being adaptable to other contexts. In addition, the method allows working with several cohorts simultaneously, while it is also possible to implement it within a single context depending on the aims of the study. Finally, the concluding step reflects the action orientation of participatory action research, as the children’s ownership of the results and their discussions with decision makers can lead to concrete actions.

## Data Availability

The datasets generated during and/or analyzed during the current study are available from the corresponding author on request.

## Abbreviations

CBPR: Community‑Based Participatory Research
KAHEQ: Kids in Action for Health Equity
PAR: Participatory Action Research
PLCP: Participant‑Led Collaborative Photovoice
UNCRC: United Nations Convention on the Rights of the Child
UNICEF: United Nations International Children’s Emergency Fund
WHO: World Health Organization
